# (Thyroid) Hormonal regulation of breast cancer cells

**DOI:** 10.3389/fendo.2022.1109555

**Published:** 2023-01-11

**Authors:** Aleck Hercbergs, Hung-Yun Lin, Shaker A. Mousa, Paul J. Davis

**Affiliations:** ^1^Department of Radiation Oncology, The Cleveland Clinic, Cleveland, OH, United States; ^2^Program for Cancer Molecular Biology and Drug Discovery, College of Medical Science and Technology, Taipei Medical University, Taipei, Taiwan; ^3^Albany College of Pharmacy and Health Sciences, Albany, NY, United States; ^4^Department of Medicine, Albany Medical College, Albany, NY, United States

**Keywords:** L-thyroxine (T4), 3, 5, 3’-triiodo-L-thyronine(T3), integrin αvβ3, breast cancer, estrogen receptor-α (ERα), euthyroid hypothyroxinemia

## Abstract

Thyroid hormone as L-thyroxine (T4) acts nongenomically at physiological concentrations at its cancer cell surface receptor on integrin αvβ3 (‘thyrointegrin’) to cause cancer cell proliferation. In the case of estrogen receptor (ERα)-positive breast cancer cells, T4 *via* the integrin promotes ERα-dependent cancer growth in the absence of estrogen. Thus, tumor growth in the post-menopausal patient with ERα-positive cancer may again be ER-dependent because of T4. Additional mechanisms by which T4 may contribute uniquely to aggressive breast cancer behavior—independently of ER—are stimulation of immune checkpoint inhibitor gene expression and of several anti-apoptosis mechanisms. These observations may call for consideration of elimination of host T4 production in breast cancer patients whose response is suboptimal to standard chemotherapy regimens. Euthyroidism in such a setting may be maintained with exogenous 3,3’,5-triiodo-L-thyronine (T3).

## Introduction

Steroid hormones have genomic and nongenomic actions that overlap, as do the genomic and nongenomic mechanisms of action of thyroid hormone (1). In the case of thyroid hormones, for example, L-thyroxine (T4) at the cell surface receptor for thyroid hormone analogues on plasma membrane integrin αvβ3 nongenomically causes phosphorylation/activation of nuclear thyroid hormone receptor TRα (2, 3). Thus, T4 that is thought to function primarily as a prohormone for nuclear 3,5, 3’-triiodo-L-thyronine (T3) can alter the activation state of the nuclear thyroid hormone receptor.

We briefly emphasize here that nongenomic action of T4 at αvβ3 may overlap with the genomic mechanism of action of estrogen at the nuclear estrogen receptor (ERα) (3). This appears to have implications for breast carcinoma cell proliferation (4) in the menopausal woman and for cancers of other tissues that may be estrogen-responsive. We also emphasize that the thyroid hormone receptor on the integrin (‘thyrointegrin’) in breast cancer cells may contribute to other clinically undesirable actions of these cancer cells, including immune checkpoint desensitization/anti-apoptosis (5).

## Thyrointegrin function in ERα-positive and –negative breast cancer cells

The proliferative effects of physiological concentrations of T4 and estradiol on ERα in human breast cancer cells are comparable and are similarly reduced by pharmacologic inhibition of mitogen-activated protein kinase (MAPK) activity (4). Activation of MAPK by T4 is thyrointegrin-dependent (6). Interestingly, integrin-dependent stimulation by T4 of proliferation of triple-negative ER cells also occurs (7). Thus, more than one mechanism exists for the stimulation by T4 of breast cancer cell proliferation. The apparent clinical implications of these observations are that 1) in post-menopausal women with ER-positive breast cancer, T4 may serve an estrogen-like function on tumor cell proliferation and 2) in pre-menopausal patients with breast cancer, T4 may be a stimulatory factor, regardless of estrogen receptor status of the disease.

## Immune checkpoint regulation *via* thyrointegrin in breast cancer cells

The programmed death-1 (PD-1)/PD-ligand 1 (PD-L1) immune checkpoint is among a set of such checkpoints that are critical cancer cell defenses against host immune system destruction by T cells (8, 9). Lin and co-workers have shown that T4 causes the accumulation in breast cancer cells—and other tumor cells—of PD-L1 and PD-1 (5) and thus may protect the cancer cells against destruction by host T cells. This pharmacologic action of T4 *via* the thyrointerin is anti-apoptotic, serving to prevent specific phosphorylation and activation of p53.

## Apoptosis inhibition by T4 *via* thyrointegrin in breast cancer cells

The preceding section introduced the topic of T4-induced anti-apoptosis *via* an immune checkpoint, but without primarily involving an immune system mechanism. In addition to disrupting pro-apoptotic activation of p53 in the intrinsic apoptotic pathway in cancer cells, T4 may call on other mechanisms to block apoptosis. For example, T4 interferes with the Fas-dependent extrinsic pathway of promoting apoptosis (5) and also enhances expression of gene coding for the X-linked inhibitor of apoptosis (XIAP) protein (5). These effects are initiated at the thyroid hormone receptor on plasma membrane integrin αvβ3. Zyla et al. have recently confirmed that T4 is anti-apoptotic in breast cancer cells (10). Thus, T4 confers on breast cancer and other tumor cells a multifactorial, anti-apoptotic behavior pattern. It is important to emphasize that it is physiological concentrations of T4 that achieve this clinically undesirable effect.

## Support by T4 of metastasis of breast cancer

A number of actions of physiological concentrations of T4 at its receptor on integrin αvβ3 on tumor cells and rapidly-dividing blood vessel cells are relevant to cancer metastasis, as we have pointed out (11). These nongenomically initiated actions culminate in regulation of expression of genes linked to angiogenesis, matrix metalloproteinases and receptor tyrosine kinases. That αvβ3 is essential to the metastasis of breast cancer to bone was shown by Sloane et al. (12) and we have reported in preclinical studies that pharmacologic blockade of this thyrointegrin eliminates established breast cancer metastases in bone and other tissues (11).

## MDR-1/P-glycoprotein gene expression, chemoresistance of breast cancer and T4

Multidrug resistance (MDR) of cancer cells is conferred by hyperexpression of single or multiple ATP binding cassettes (ABC) transporters that, located in the cell nucleus, export chemotherapeutic agents that target the nucleus (13). MDR in breast cancer cells is particularly a function of expression of *ABCB1* (MDR-1/p-glycoprotein, P-gp) (13, 14). Thyroid hormone causes transcription of P-glycoprotein (15, 16) and also stimulates activity of the transcript (17). We have shown that tetrac can restore chemosensitivity of tumor cells (18) and thus we propose that the action of T4 to stimulate transcription of the *ABCB1* gene—and support chemoresistance—is mediated by integrin αvβ3.

## Discussion: Clinical implications of actions of T4 on breast cancer cells

The purpose of this brief review is to suggest that thyroid hormone as T4 has actions that may be sources of difficulties in management of breast cancer. Examples presented here are T4 acting like an estrogen at ER in the postmenopausal patient, induced failure by T4 of immune checkpoint inhibitor drugs in management of breast cancer patients and T4-induced suboptimal effectiveness of pro-apoptotic regimens.

We know that the risk of developing breast cancer is increased in hyperthyroidism (19, 20) and somewhat decreased in hypothyroidism (10), that levothyroxine increases the clinical risk of breast cancer (21) and that hypothyroidism can alter the clinical course of breast cancer for the better (22). In addition, the substitution of T3 for T4 in maintaining euthyroidism (euthyroid hypothyroxinemia) can improve the course of management of advanced cancers, including breast cancer (23). Given such information and the ability of T4 to promote breast cancer cell proliferation *in vitro*, we propose that 1) euthyroid hypothyroxinemia be considered a therapeutic option in euthyroid breast cancer patients whose response to conventional anti-breast cancer therapy is suboptimal and 2) this stratagem be prospectively studied.

The reported actions of thyroid hormone on P-gp gene expression, whether genomic (15) or nongenomic (24), raise the possibility that the hormone in the cancer patient may foster tumor cell chemoresistance. This possibility has not yet been examined in breast cancer cells, but integrin αvβ3—which bears the cell surface receptor for T4—has been shown to be an important contributor to the doxorubicin resistance of metastatic breast cancer (14). Breast cancer cell chemoresistance may also be a consequence of XIAP gene expression (25) and we have noted above that T4, acting *via* its plasma membrane receptor, upregulates expression of the gene for this factor.

## Author contributions

The initial draft of the manuscript was written by PD, but all co-authors edited the drafts. This is a review manuscript and all authors have published multiple pre-clinical or clinical observations that are cited in the References section. All authors contributed to the article and approved the submitted version.

## References

[1] Hammes SR Davis PJ. Overlapping nongenomic and genomic actions of thyroid hormone and steroids. Best Pract Res Clin Endocrinol Metab (2015) 29(4):581–93. doi: 10.1016/j.beem.2015.04.001 PMC4976293 26303085

[2] Cheng SY Leonard JL Davis PJ. Molecular aspects of thyroid hormone actions. Endocr Rev (2010) 31:139–70. doi: 10.1210/er.2009-0007 PMC2852208 20051527

[3] Davis PJ Mousa SA Lin HY. Nongenomic actions of thyroid hormone: The interin component. Physiol Rev (2021) 101:319–52. doi: 10.1152/physrev.000328.2019 32584192

[4] Tang HY Lin H-Y Zhang S Davis FB Davis PJ. Thyroid hormone causes mitogen-activated protein kinase-dependent phosphorylation of the nuclear estrogen receptor. Endocrinology (2004) 145:3265–72. doi: 10.1210/en.2004-0308 15059947

[5] Lin HY Chin YT Shih YJ Chen YR Leinung M Keating KA. In tumor cells, thyroid hormone analogues non-immunologically regulate PD-L1 and PD-1 accumulation that is anti-apoptotic. Oncotarget (2018) 9:34033–7. doi: 10.18632/oncotarget.26143 PMC6183344 30344919

[6] Bergh JJ Lin HY Lansing L Mohamed SN Davis FB Davis PJ. Integrin alphavbeta3 contains a cell surface receptor site for thyroid hormone that is linked to activation of mitogen-activated protein kinase and induction of angiogenesis. Endocrinology (2005) 146:2864–71. doi: 10.1210/en.2005-0102 15802494

[7] Hercbergs A Mousa SA Leinung M Lin HY Davis PJ. Thyroid hormone in the clinic and breast cancer. Horm Cancer (2018) 9:139–43. doi: 10.1007/s12672-018-0326-9 PMC5945724 29441459

[8] Alsaab HO Sau S Alzhrani R Tatiparti K Bhise K Kashaw SK. PD-1 and PD-L1 checkpoint signaling sinhibition for cancer immunotherapy: Mechanism, combinations, and clinical outcome. Front Pharmacol (2017) 8:561. doi: 10.3389/fpharm.2017.00561 28878676 PMC5572324

[9] Vareki SM Garrigos C Duran I. Biomarkers of response to PD-1/PD-L1 inhibition. Crit Rev Oncol Hemtol (2017) 116:116–24. doi: 10.1016/j.critrevvonc.2-17.06.001 28693793

[10] Zyla LE Cano R Gomez S Escudero A Rey L Santiano FE. Effects of thyroxine on apoptosis and proliferation of mammary tumors. Mol Cell Endocrinol (2021) 538:111454. doi: 10.1016/j.cme.2021.111454 34520813

[11] Mousa SA Glinsky GV Lin H-Y Ashur-Fabian O Hercbergs A Keating KA. Contributions of thyroid hormone to cancer metastasis. Biomed (2018) 6:89. doi: 10.3390/biomedicines6030089 PMC6165185 30135398

[12] Sloane EK Poulio M Stanley KL Chia J Moseley JM Hards DK. Tumor-specific expression of αvβ3 integrin promotes spontaneous metastasis of breast cancer to bone. Breast Cancer Res (2006) 8:R20. doi: 10.1186/bcr1398 16608535 PMC1557720

[13] Kabori T Harada S Nakamoto K Tpkuyama S. Mechanisms of p-glycoprotein alteration during anticancer treatment: Role of the pharmacokinetic and pharmacological effects of various substrate drugs. J Pharmacol Sci (2014) 125(3):242–54. doi: 10.1254/jphs.14r01cr 24989947

[14] Bao L Hazari S Mehra S Kaushal D Moroz K Dash S. Increased expression of p-glycoprotein and doxorubicin chemoresistance of metastatic breast cancer is regulated by miR-298. Am J Pathol (2012) 180(6):2490–503. doi: 10.1016/j.ajpath.2012.02.024 PMC3378910 22521303

[15] Nishio N Katsura T Inui K-I. Thyroid hormone regulates the expression and function of p-glycoprotein in caco-2 cells. Pharm Res (2008) 25(5):1037–42. doi: 10.1007/s1109-007-9495-x 18004648

[16] Kurose K Saeki M Tohkin M Hasegawa R. Thyroid hormone receptor mediates human MDR1 gene expression – identification of the response region essential for gene expression. Arch Biochem Biophys (2008) 474(1):82–90. doi: 10.1016/j.abb.2008.03.020 18395509

[17] Burk O Brenner SS Hofmann U Tegude H Igel S Schwab M. The impact of thyroid disease on the regulation, expression and function of ABCB1 (MDR1/P-glycoprotein) and consequences of the disposition of digoxin. Clin Pharmacol Ther (2010) 88(5):685–94. doi: 10.1007/s10456-007-9088-7 20844484

[18] Rebbaa A Chu F Davxis FB Davis PJ Mousa SA. Novel function of the thyroid hormone analogue tetraiodothyroacetic acid: A cancer chemosensitizing and anti-cancer agent. Angiogenesis (2008) 11(3):269–76. doi: 10.1038/clpt.2010.176 18386142

[19] Sogaard M Farkas DK Ehrenstein V Jorgensen JOL Dekkers OM Sorensen HT. Hypothyroidism and hyperthyroidism and breast cancer risk: a nationwide cohort study. Eur J Endocrinol (2016) 174(4):409–14. doi: 10.1530/EJE-15-0989 26863886

[20] Khan SR Chaker L Ruiter R Aerts JGVA Hofman A Dehghan A. Thyroid function and cancer risk: The Rotterdam study. J Clin Endocrinol Metab (2016) 101(12):5030–6. doi: 10.1210/jc.2016-2104 27648963

[21] Wu C-C Yu Y-Y Yang H-C Nguyen PA Poly TN Islam MM. Levothyroxine use and the risk of breast cancer: A nation-wide population-based case-control study. Arch Gynecol Obstet (2018) 298(2):389–96. doi: 10.1007/s00404-018-4837-y 29961136

[22] Cristofanilli M Yamamura Y Kau SVV Bevers T Strom S Patangan M. Thyroid hormone and breast carcinoma. primary hypothyroidism is associated with a reduced incidence of primary breast cancer. Cancer (2005) 103:1122–8. doi: 10.1002/cncr.20881 15712375

[23] Hercbergs A Johnson RE Ashur-Fabian O Garfield DH Davis PJ. Medically-induced euthyroid hypothyroxinemia may extend survival in compassionate need cancer patients: an observational study. Oncologist (2015) 20:72–6. doi: 10.1634/theoncologist.2014-0308 PMC4294612 25410096

[24] Davis PJ Incerpi S Lin HY Tang HY Sudha T Mousa SA. Thyroid hormone and p-glycoprotein in tumor cells. BioMed Res Int (2015) 2015:168427. doi: 10.1155/2015/168427 25866761 PMC4383522

[25] Delbue D Mendonca BS Robaina MC Lemos LGT Lucena PI Viola JPB. Expression of nuclear XIAP associated with cell growth and drug resistance and confers poor prognosis in breast cancer. Biochim Biophys Acta (BBA) – Mol Cell Res (2020) 1867(10):118761. doi: 10.1016/j.bbamcr.2020.118761 32485270

